# Supplementary material for: Insular functional organization during handgrip in females and males with obstructive sleep apnea
Source: PLoS One. 2021 Feb 18;16(2):e0246368. doi: 10.1371/journal.pone.0246368 (PMC7891756; doi:10.1371/journal.pone.0246368)
Supplement: S1 File — (DOCX) [file pone.0246368.s001.docx]

**S1 File: screening**

**Phone screening questions**

Now I’m going to ask you a series of questions. Please do not answer the following four questions individually. When I am done asking them, you may say “yes” if one or more of the questions in the group apply.

1. Do you have a previous history of cardiac disease, stroke, vascular congenital anomalies?
2. Are you diabetic?
3. Do you have or have you ever experienced any claustrophobia (i.e., abnormal dread of being in closed or narrow spaces)?
4. If you are female, are you pregnant?
5. (Control) Do you experience now, or have you ever experienced any severe or debilitating sleep problems, including sleep apnea? (OSA) Do you experience now, or have you ever experienced any severe or debilitating sleep problems, NOT including sleep apnea?
6. Are you taking or have you ever taken any mood-altering drugs (anti-depressants, such as Prozac) or any recreational drugs (marijuana, cocaine, etc.)?

Do one or more of these questions in the group apply?

If YES, the person is not eligible.

**In-person questions for semi-structured interview of potential healthy control participants**

In order to screen for potential OSA, these topics are addressed during a conversation with a research team member at the first visit to UCLA, prior to describing the study procedures and consent process. If a potential participant’s answers suggest possible undiagnosed OSA, they were recommended to contact their primary care physician to discuss receiving a sleep study.

- Snoring with gasping
- Waking feeling out of breath
- Partner mentioning stopping breathing, or struggling to breath during sleep
- Waking feeling excessively tired even after many hours (>8) in bed, and excessive tiredness during the day
