# Supplementary material for: Insular functional organization during handgrip in females and males with obstructive sleep apnea
Source: PLoS One. 2021 Feb 18;16(2):e0246368. doi: 10.1371/journal.pone.0246368 (PMC7891756; doi:10.1371/journal.pone.0246368)
Supplement: Attachment — Submitted filename: PLOS ONE response to reviewers-v2.docx [file pone.0246368.s003.docx]

*The Introduction should be further developed to explain to readers about the relevance of handgrip for OSA patients. What is the point of handgrip? By reading through the current version of the Introduction, it is difficult for the reviewer to understand the connection or relevance between the two. Afterwards the text should explain why sexual differences in cerebral processing of handgrip in OSA patients are important. Why not foot grip or intentional forceful exhalation?*

We have extensively revised the introduction to remove some jargon, better explain the rationale for performing the handgrip, and to contrast this task with other autonomic challenges.

*From the Figures reporting fMRI time trend results, it seems that the differences were very subtle. Please further explain to the readers the significance of differences with such a small magnitude.*

We added a short paragraph in the discussion address this issue, and clarify two points regarding the findings:

1) fMRI signal changes in general to any task are in the order of 1%, and often less, so the patterns we observed of 0.3 to 1 % change are typical for this modality. In most neuroimaging papers, the magnitude of fMRI signal changes is not shown so our presentation is unusual. However, we believe there is value in showing the ready the actual signal changes underlying the statistical findings.

2) We presented both positive and negative findings. Many of the comparisons unexpectedly showed no significant difference between OSA and control, and hence the timetrends often reflect a lack of change. Furthermore, some regions (the more posterior insula, for example) show little response, and these small responses are a scientifically interesting contrast to other areas (the mid-short gyrus, for example).

*There are multiple neuroimaging meta-analysis of OSA patients in terms of morphometric, functional connectivity and activation changes (c.f. 10.1016/j.neubiorev.2016.03.026 ; 10.1111/jsr.12857 ; 10.2147/NDT.S161085 ; just to name a few as examples). Did the current results show spatial convergence with brain regions reported in previous meta-analysis? The Discussion is infertile without references to and comparison with the multiple meta-analysis.*

We thank the reviewer for pointing out these articles, and we now refer to them in the Introduction and Discussion. They help make the case that the insula within a whole-brain network shows OSA-related functional differences.

To enable comprehensive comparison with other studies, we now added the VOI as a supplementary data (S2). With regards linking to results of these meta analyses, we clarified that we are looking at within-structure patterns only. Thus, the present findings can be compared with existing meta-analysis that refer to the insula. The sub-regions can be assessed using the VOI files.

*Reviewer #2: 

1) The sex based analysis seems to be a second thought as expressed by the authors in the discussion. This is evident from the fact that in the methods it is not clear how the authors will look at sex differences. In the introduction (line 76) the authors state that they will control for sex differences. This means looking at the brain activations above and beyond the effects of sex. However the goal in the paper was to look at different mechanisms in males and females which would be better done with stratified analysis, which I believe the authors actually did. In light of this I suggest to re write the last paragraph of the introduction and methods to clearly state that the main interest is Sex differences and that separate analyses in males and females will be conducted to assess them.*

We reworded the Introduction to reflect what we did; the term “control” was meant to refer only to the mixed-sex analysis, and this is now explicitly stated in the last paragraph of the Introduction. We also clarified the Abstract and Methods.

*2) Furthermore, in the introduction the authors explain how sex differences may be due to hormonal effects that impact both susceptibility to OSA and sympathetic responses. Did the authors collect information about menstrual cycle, menopause (average age for females in 50), use of hormonal replacement therapy? If this information was not collected this should be discussed as a limitation.*

This information was unfortunately not collected. We added a note in the limitations.

*3) More details are needed about how these participants were diagnosed for OSA. Did they undergo a full level 2 polysomnography or just an apnea test? Moreover more information about sleep quality and other sleep parameters should be reported ( i.e. sleep efficiency, awakenings etc.). The authors mention a semi structured interview to assess symptoms of OSA. This information should be reported.*

We clarify that OSA patients were diagnosed based on a full in-clinic sleep study. Our sleep study data were based off sleep reports provided, and did not typically include other metrics; we note this in the limitations. We added the phone screening and guide to the semi-structured in-person interview as a supplementary file (S1).

*4) In table 1, the authors report AHI values that I believe were assessed by one of the aforementioned methods but then report the oxygen saturation collected in the scanner. This is unclear. Please report oxygen desaturation index and mean saturation through the sleep study night. Moreover mean saturation (whether in the scanner or during sleep) is not really useful because can indicate only a dip in saturation. Mean saturation would be more useful.*

We addressed this confusion. The in-scanner signal was only used to calculate heart rate, and a pulse oximeter can be MRI-compatible since the sensor is based on light transmission. We clarified Table 1, including mean O_2_.

*5) What is the reason to use VBM 8 based on DARTEL ? Where you expecting structural abnormalities, atrophy in these patients. Please specify.*

We clarified this aspect of the analyses; we used the DARTEL normalization to the VBM8 template to align the subjects.

*6) Please report MNI coordinates for the different ROIs.*

We now provide the ROI as a supplementary file, so all voxels are included.

*7) Previous studies reported differential activation of the insula according to individuals sleep quality (Guadagni et al. 2018,* [*https://doi.org/10.1111/ejn.14124*](https://doi.org/10.1111/ejn.14124)*). Sleep quality should be used as a covariate in the models or at least this should be discussed as a possible confounder.*

Unfortunately, we did not have a measure of sleep quality in the sleep reports. We did have a measure of OSA severity in terms of the AHI, but this measure is notoriously poor at predicting either symptoms or pathophysiology. Furthermore, we did not have sleep reports on the control group. We now mention this factor in the limitations, and refer to the interesting Guadagni article.

*8) The discussion should be re organized. The findings should be discussed in the first paragraph then connected to existing literature.*

We agree the original Discussion missed an overview of the findings, and we have revised accordingly.

*Minor:
- Why is line 151 italicized?*

We have corrected this and other formatting, including changes in text to match the journal requirements.

*- Table 2: please report that the values are p values either in the legend. It took me a while to see it in the table which is perhaps a little busy.*

We revised the legend and added text in the table; we also revised legends for Tables 3-5.
